# Serum Levels of Interleukin-6 and Interleukin-10 as Biomarkers for Hepatocellular Carcinoma in Egyptian Patients

**DOI:** 10.1155/2013/412317

**Published:** 2013-09-15

**Authors:** Mohamed S. Othman, Ahmed M. Aref, Amal A. Mohamed, Wesam A. Ibrahim

**Affiliations:** ^1^Biochemistry Department, Faculty of Pharmacy, October University for Modern Sciences and Arts (MSA), Giza 11787, Egypt; ^2^Biology Department, Faculty of Dentistry, October University for Modern Sciences and Arts (MSA), Giza 11787, Egypt; ^3^Biochemsitry Department, National Hepatology and Tropical Medicine Research Institute, Fom El-Khalig, Cairo 11796, Egypt; ^4^Faculty of Medicine, Ain Shams University, Cairo, Egypt

## Abstract

Interleukin-10 (IL-10) and interleukin-6 (IL-6) have been reported to be related to hepatocellular carcinoma (HCC) prognosis. This study aimed to investigate the clinical usefulness of serum levels of IL-6 and IL-10 as biomarkers for HCC among high-risk patients. *Materials and Methods*. 80 individuals were enrolled in this study; they were categorized into 4 groups: group 1 healthy individuals (NC) (*n* = 20), group 2 chronic hepatitis C virus (HCV) patients (*n* = 20), group 3 cirrhotic patients (LC) (*n* = 20), and HCC group (*n* = 20). Using ELISA technique serum levels of IL-6, IL-10, and alpha fetoprotein (AFP) were evaluated in all groups. *Results*. The mean serum levels of IL-6 were significantly higher in HCC than in LC, HCV, and NC groups (13.99 ± 1.80, 7.49 ± 0.43, 5.78 ± 0.74, and 2.57 ± 0.31), respectively (*P* < 0.05); also the serum levels of IL-10 were significantly higher in HCC compared with LC, HCV, and NC groups (13.69 ± 1.89, 7.37 ± 0.53, 5.18 ± 0.6, and 3.31 ± 0.42) (*P* < 0.05). We also found that the tumor size is correlated strongly with IL-6 and IL-10 levels (*r* = 0.925, *P* < 0.001; *r* = 0.821, *P* < 0.001), respectively. *Conclusion*. The combination of those markers may help to identify a group of HCC patients with low AFP.

## 1. Introduction

Hepatocellular carcinoma is one of the most common malignant tumors, representing more than 5% of all cancers. The estimated annual number of cases exceeds 500,000, with a mean annual incidence of around 3-4%. In terms of relative frequencies, HCC ranks as the fifth most common cancer in the world; it is also the fifth among men and eighth among women; it is the second among cancers of the digestive tract after stomach cancer [1]. 

The main risk factors for HCC are hepatitis B virus (HBV), HCV, alcohol, aflatoxin, and possibly obesity and diabetes. Together, HBV and HCV account for 80% to 90% of all cases of HCC worldwide [2, 3]. 

Egypt has possibly the highest HCV prevalence in the world; 10%–20% of the general population is infected and HCV is the leading cause of HCC and chronic liver disease in the country. Approximately 90% of Egyptian HCV isolates belong to a single subtype, 4a, which responds less successfully to interferon therapy than other subtypes [4, 5]. The burden of HCC has been increasing in Egypt with a doubling in the incidence rate in the past 10 years [6]. 

The single most important tumor marker for HCC is AFP. HCC surveillance with serum AFP level and ultrasonography has been recommended for patients with cirrhosis. Although the detection of serum AFP level is well established in the screening and diagnostic purpose for HCC, a major shortcoming is that serum AFP is insensitive for the early cancer detection [7, 8]. 

In a prospective study by Marrero [9], they showed that the AFP as a surveillance tool indicates a sensitivity of 41–60%, specificity of 80–94%, and a positive predictive value of 9–32% for early HCC. 

Given improvement in the overall survival of patients with cirrhosis and the continued rise in the incidence of HCC in Egypt, strategies for the detection of early HCC in the at-risk population may lead to improved survival of patients with this deadly tumor [10]. 

The poor sensitivity of AFP renders it unsatisfactory for this purpose and suggests the need for novel biomarkers for the detection of early HCC [6]. Several biomarkers, such as des-gamma carboxyprothrombin, human hepatocytes growth factor, and insulin-like growth factor-1 are promising, but none of these markers has been validated for clinical use [11]. 

 Mounting evidence indicates the involvement of cytokines in hepatocarcinogenesis [12, 13]. IL-6 is a multifunctional cytokine. Serum IL-6 levels are elevated in patients with chronic liver inflammation including alcoholic hepatitis, HBV, HCV infections, and steatohepatitis. Many studies indicated a big role for IL-6 in the process of liver damage and carcinogenesis [14, 15]. 

 IL-10 is a pleiotropic cytokine produced by macrophages, T-helper 2 (Th2) cells and B-lymphocytes, and both can stimulate and suppress the immune response. IL-10 has been shown to inhibit various immune reactions [16]. 

Recently, it has been proposed that IL-10 plays a key role in the oncogenetic and metastatic ability of neoplasms [17]. Increased circulating IL-10 has been reported in patients with different types of tumors including resectable HCC [8, 11]. 

Serum IL-10 concentration has been reported to be significantly elevated in patients with chronic HCV and IL-10 may be related to hepatocarcinogenesis with suppression of immune surveillance [18]. 

Hsia et al. [17] reported that both IL-6 and IL-10 levels were frequently elevated in patients with HCC and that both have been reported to be related to the disease prognosis in HCC.

The aim of this study is to investigate and analyze the discriminate power of serum IL-6 and IL-10 individually and whether their combination with AFP would increase accuracy in discriminating Egyptian patients with HCC from healthy and cirrhotic subjects. Up to our knowledge a similar study was not done before in Egypt.

## 2. Materials and Methods

Eighty individuals were included in this study. They were selected from Tropical and Internal Medicine Department in a big medical center in Cairo.

This study comprises the following groups. 

*Group 1*. Normal control (NC) group, including 20 apparently healthy volunteers (10 males and 10 females, their age range: from 33 to 77 years). They are negative for HCV-Ab and have normal abdominal ultrasonography with no evidence of liver disease and/or of neoplasm. 

*Group 2*. HCV group, including 20 patients (13 males and 7 females, their age range: from 33 to 71 years). They are infected with HCV (positive for both HCV-Ab and HCV RT-PCR). 

*Group 3*. Cirrhotic (LC) group, including 20 patients (15 males and 5 females, their age range: from 32 to 78 years). They are suffering from liver cirrhosis with no histological evidence of cancer. 

*Group 4*. HCC group, including 20 patients (12 males and 8 females, their age range: from 35 to 76 years). HCC was confirmed by pathology, imaging (computer tomography (CT) and ultrasound), and serum AFP. Tumor size was measured according to CT imaging.

All the studied groups included in this study were free from heart diseases, kidney diseases, muscle disorders, pancreatitis and bilharziasis, and HBV. Also patients with special habits like smokers and alcoholics were excluded from this study to prevent any external interference.

All patients and controls gave their informed consent which was ethically conducted in accordance with the Helsinki Declaration. 

Ten milliliters of fasted venous blood were taken from patients and controls; serum was separated by centrifugation and stored at −80°C until further examinations.

 All patients were subjected to the following.

- Clinical assessment: all the patients were subjected to abdominal ultrasound, liver biopsy, and laboratory assessment.
- Laboratory evaluation including
  - hepatitis C virus: anti-HCV antibodies by third generation ELISA (Sorin Biomedica Diagnostics, Italy) [19] and RT-PCR for HCV; nucleic acid extraction was done by QIAGEN viral RNA Mini-Extraction Kit [20];
  - hepatitis B virus: HbsAg and anti-HBc were performed by a direct noncompetitive sandwich assay (DiaSorin, Italy) based on ELISA technique [21].
- IL-10 and IL-6 and AFP were detected using a third generation ELISA (WKEA Med Supplies Corp., Changchun, China) [22].
- Liver function tests: A diazotization method used for determination of serum total bilirubin [23]. Activities of ALT and AST were measured by the enzyme rate method [24]. Albumin was determined according to Pinnell and Northam [25] method. Prothrombin time was determined using standard thromboplastin method [26].
- Blood picture was done on Coulter Counter T890, (Coulter Counter, Harpenden, UK) [27].
- Histological studies: HCC neoplastic cells were identified histopathologically in H&E-stained sections of a core needle biopsy.

All tests were done according to the manufacturer's instructions.

Data were analyzed by SPSS statistical package version 19. One-way ANOVA, Duncan multiple comparisons, and Pearson's correlation coefficient were used for analysis. Multiple receiver operating characteristic curves (ROC curves) were drawn to assess the validity of the tumor markers. A *P* value < 0.05 was considered significant [28].

## 3. Results

Table 1 demonstrates the number, age, and the results of different biochemical parameters carried out in this study including different liver function testes as well as different parameters of blood picture of individuals from all investigated groups.

Table 2 illustrates the mean levels of serum of IL-6, IL-10, and AFP as well as their range in the studied groups. Significant elevations were observed in the levels of IL-6, IL-10, and AFP in the disease groups, giving a tremendous increase in the HCC group.

As shown in Table 2, the mean serum levels of AFP were significantly higher in HCC, LC, and HCV (255.8 ± 48.43, 21.25 ± 5.97, and 13.51 ± 3.27) than NC group (3.34 ± 0.41) (*P* = 0.001). The serum levels of AFP were significantly elevated in HCC group compared with both HCV group and LC group (*P* = 0.01); there was also a significant increase in AFP level in LC group as compared with HCV group (*P* < 0.05). 

The serum levels of IL-6 were significantly higher in HCC, LC, and HCV groups (13.99 ± 1.80, 7.49 ± 0.43, and 5.78 ± 0.74) than NC group (2.57 ± 0.31) (*P* = 0.001), and there is a consistent increase in the IL-6 level with the disease progression from NC to HCC. The serum levels of IL-6 were significantly elevated in HCC group compared with both HCV group and LC group (*P* < 0.05) but no significant difference in IL-6 level between HCV group and LC group.

 The serum levels of IL-10 were significantly higher in HCC, LC, and HCV groups (13.69 ± 1.89, 7.37 ± 0.53, and 5.18 ± 0.6) than NC group (3.31 ± 0.42) (*P* = 0.001). The serum levels of IL-10 were significantly elevated in HCC group compared with both HCV and LC groups (*P* < 0.05); however, there was no significant difference in IL-10 level between HCV group and LC group.

Table 3 shows the AUC, cutoff values, sensitivities, and specificities positive predictive value (PPV) and negative predictive value (NPV) of IL-6, IL-10, and AFP. The best sensitivities and specificities for the benign liver diseases taken collectively versus the HCC group were achieved by IL-6, IL-10, and AFP. 

There was a significant association between IL-6 and IL-10 levels in both LC and HCC groups (*r* = 0.676, *P* = 0.001; *r* = 0.846, *P* = 0.001), respectively. In HCC patients tumor size was correlated strongly with IL-6 and IL-10 levels (*r* = 0.925, *P* = 0.001; *r* = 0.821, *P* = 0.001), respectively (Figures 4 and 5). 

In HCV group there was a significant correlation between AFP and IL-6 (*r* = 0.64, *P* = 0.002). But there was no type of correlations between serum IL-6 or IL-10 and serum AFP in HCC and LC groups.

Receiver operating characteristic (ROC) curves were plotted to define the optimal cutoff values and to identify the specificity and sensitivity for serum AFP, IL-6, and IL-10 in differentiating HCC from benign liver diseases (patients with HCV, LC, and normal control) (Table 3).

At serum level 20 ng/mL (recommended cutoff value for AFP which is mainly used worldwide), the AUROC for AFP was 0.76 (95% CI: 56.3–94.1), with a sensitivity of 80%, specificity 80%, and accuracy 80%. 

**Figure 1:**
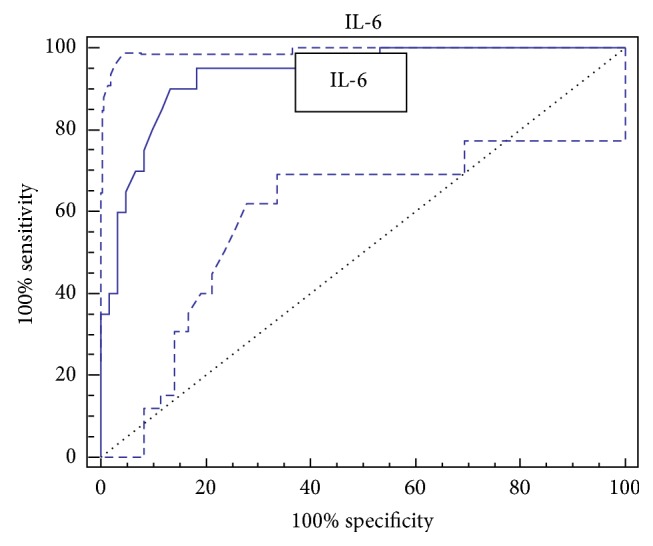
ROC curve of IL-6 for the diagnosis of HCC.

**Figure 2:**
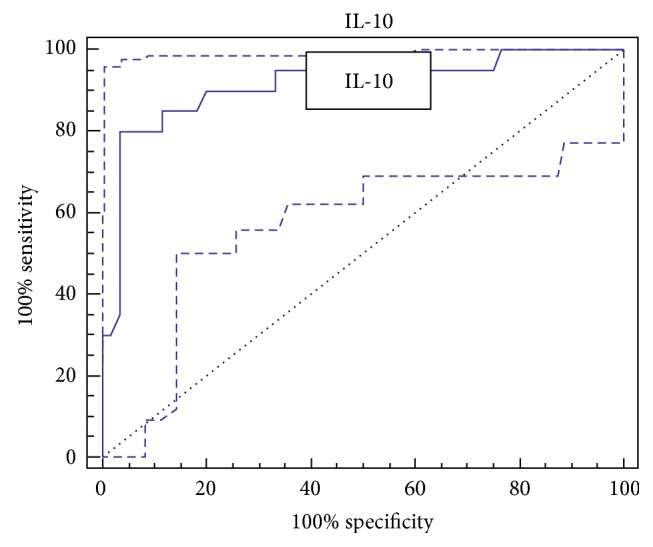
ROC curve of IL-10 for the diagnosis of HCC.

 The AUROC for IL-6 was 0.931 (95% CI: 0.851 to 0.975), with a sensitivity of 90%, specificity 86.67%, accuracy 87.5%, and optimal cutoff value 8.6 pg/mL (Figure 1). The AUROC for IL-10 was 0.914 (95% CI: 0.829–0.965), with a sensitivity of 80%, specificity 96.67%, accuracy 92.5%, and optimal cutoff value 9.7 pg/mL (Figure 2). 

**Figure 3:**
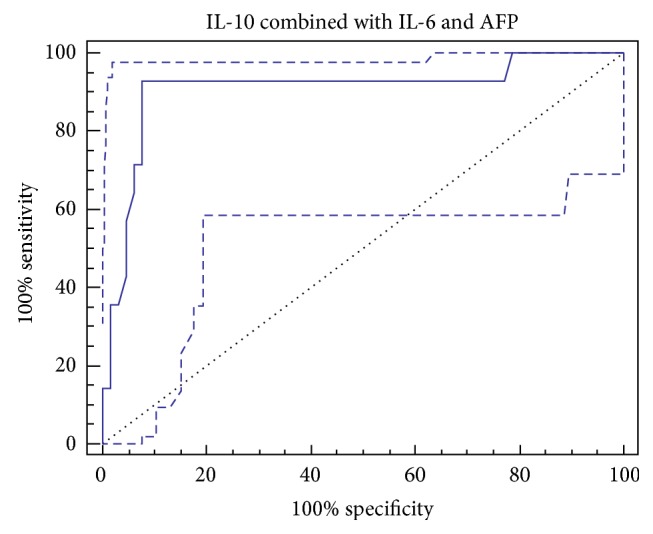
ROC curve of IL-10 combined with IL-6 and AFP for the diagnosis of HCC.

**Figure 4:**
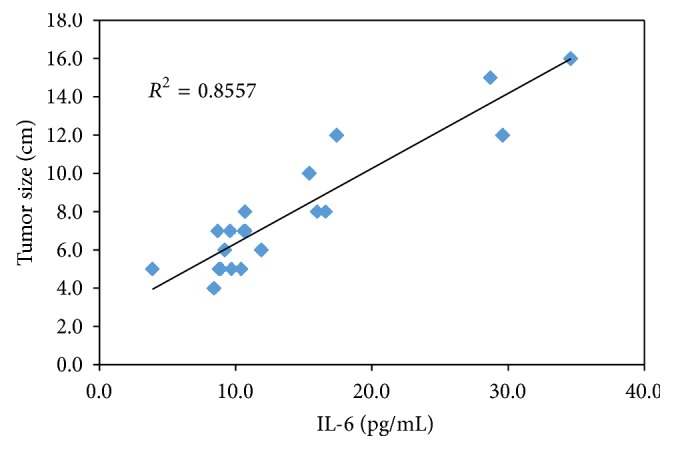
Correlation between the serum IL-6 and tumor size in the HCC group.

**Figure 5:**
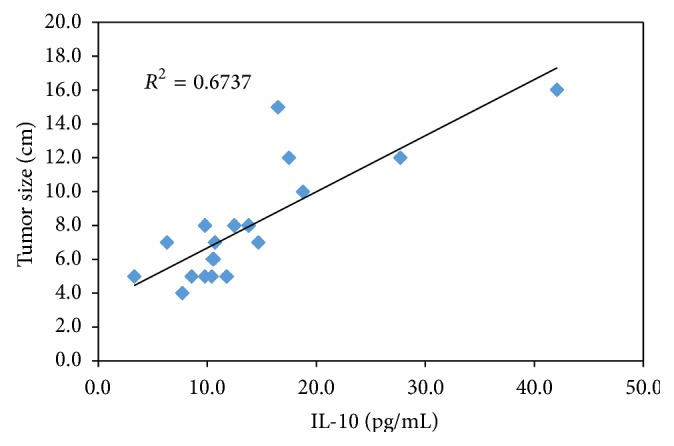
Correlation between the serum IL-10 and tumor size in the HCC group.

The values for the combined detection using more than one marker are also presented in Figure 3. The detection using a combination of IL-10 with IL-6 and AFP produced better sensitivity (92%) and specificity (92.3%).

## 4. Discussion

HCC is responsible for a large proportion of cancer deaths worldwide. HCC is frequently diagnosed after the development of clinical deterioration at which time survival is measured in months. Long-term survival requires detection of small tumors, often present in asymptomatic individuals, which may be more amenable to invasive therapeutic options. Surveillance of high-risk individuals for HCC is commonly performed using the serum marker AFP often in combination with ultrasonography. Serum AFP used alone can be helpful if levels are markedly elevated, which occurs in fewer than half of cases at time of diagnosis [29, 30]. 

This study revealed a significant increase in AFP serum levels in HCC and some benign liver diseases such as hepatitis and cirrhosis. AFP was significantly lower in benign liver diseases as compared with HCC. Similar results were obtained by many studies [30–32]. In the current study, the sensitivity, specificity, and accuracy of AFP were 80%, 80%, and 80% at the cutoff level of 20 ng/mL (recommended cutoff value for AFP which is mainly used worldwide) [33–35]. Our results were in agreement with Abdel-Haleem et al. [36]. 

 In the present study we found that 20% (4/20) of HCC patients had serum AFP level <20 ng/mL. The diagnosis of HCC could be very difficult in high-risk patients when these patients had low AFP level or tumors mimicking HCC. It has been estimated that up to 75% of patients with small HCC and 20% of patients with large HCC may have normal serum AFP level that could escape from HCC surveillance [37, 38]; however, several investigators concluded that AFP fails as a reliable marker, mainly because it shows poor sensitivity and specificity [7, 39].

 Both IL-6 and IL-10 are multifunctional cytokines produced by a range of cells and play a central role in host defense mechanism and modulation of immune response [40]. An increasing body of evidence indicates a key role of IL-6 and IL-10 in the process of liver damage and carcinogenesis. These two cytokines have been implicated to associate with certain human cancers and HCC [41]. 

The results of the present study showed that serum levels of IL-6 were significantly higher in all patients groups compared with control group, and we found significantly higher circulating IL-6 titers in HCC than in both cirrhotics and HCV groups. 

Our findings were in agreement with the results of other studies, Porta et al. [32] Hsia et al. [17] Malaguarnera et al. [42], Maurizio et al. [43], and Ataseven et al. [44]. They showed that IL-6 levels in patients, with HCC are higher than those in LC, chronic HCV patients, and controls. 

Our results were not consistent with Zekri et al. [20] and Tovey et al. [45], who showed that IL-6 was slightly higher only in asymptomatic HCV carriers than controls but apparently normal in both HCC and chronic liver disease (CLD) patients. Our results were not consistent also with Metwaly et al. [46], who found a significant decrease in serum IL-6 concentration in HCC patients as compared to patients with liver cirrhosis.

There are many reasons to make IL-6 as intriguing cytokine to study in HCC patients. Naugler and Karin [16] and Jin et al. [47] suggested that chronic exposure to high IL-6 level is associated with increased liver injury and HCC development in animals. This was in line with the study of Wong et al. [48], who confirmed that patients who subsequently developed HCC had raised IL-6 levels 2-3 years before HCC development. 

IL-6 was also shown to induce the expression of the mitogenic, morphogenic, and proneoangiogenic scatter factor hepatocyte growth factor which, besides being commonly expressed at high levels in HCCs, also signals using the same STAT-3 pathway as IL-6 [49, 50].

Porta et al. [32] reported that transfection of IL-6 into nonmetastatic HCC cells makes them highly metastatic. Liu et al. [51] reported that IL-6 promoted survival of human liver cancer cells through activating STAT-3 in response to doxorubicin treatment.

IL-6 may also decrease HCC cell apoptosis, thus conferring a survival advantage to the tumor; indeed, in a mouse model, IL-6 proved to reduce Fas-induced apoptosis [52]. IL-6 may directly stimulate hepatic DNA synthesis, since IL-6 transgenic mice showed a lack in DNA synthesis following hepatectomy, and double transgenic mice expressing both IL-6 and soluble IL-6 receptor (sIL-6R) under a liver-specific promoter develop hepatocellular hyperplasia and adenomas, which are considered as precancerous lesions in humans [52, 53].

IL-6 has also been proposed as a cause of natural killer cell dysfunctions, thus potentially representing a mechanism of tumor escape from immune surveillance [41].

In this study we found an elevated level of circulating IL-10 in patients with HCV, cirrhosis, and HCC and the concentrations are associated with disease progression.

These results were in agreement with Hattori et al. [54], who found that serum IL-10 levels are significantly higher in all chronic liver disease groups (HCV, LC, and HCC) than in controls, indicating that IL-10 reflects the degree of inflammation in the liver and may be related to the development of HCC.

IL-10 cytokine is an anti-inflammatory endogenous mediator through the inhibition of the production of proinflammatory cytokines. Zekri et al. [13] reported that the high serum IL-10 levels in patients with HCC result from the secretion of IL-10 by tumor cells, in addition to the production at the site of inflammatory changes with activated infiltrating mononuclear cells in the liver.

 The immunosuppressive effects of IL-10 may play a major role in the development of neoplastic processes by suppressing macrophage activation and interferon-gamma production, thereby crippling two potential mediators of an antitumor response; this may help the tumor cells escape host immune surveillance and potentiate tumor cells to metastasize.

 The functional consequences of IL-10 binding to its receptors on tumor cells could be the prevention of programmed cell death and the promotion of proliferation. In addition, IL-10 may contribute to the development of an environment favorable to neoplastic cells and to the enhancement of their metastatic potential [37]. 

In the current study, the sensitivity, specificity, and accuracy of AFP, IL-6, and IL-10 were 80%, 80%, and 80%; 90%, 86.67%, and 87.5%; 80%, 96.67%, and 92.5%, respectively. 

Using the optimal cutoffs derived from the ROC curves IL-6 level was elevated in 3 patients out of 4 who were with AFP <20 ng/mL, while IL-10 level was elevated in 2 of them. Our results indicated a potential role for IL-6, and IL-10 as a tumor marker for HCC with respect to AFP and this was in agreement with the previous studies [14, 17, 31, 32, 37]. 

 Using the optimal cutoffs derived from the ROC curves a combination of IL-10, IL-6, and AFP produced better sensitivity (92%) and specificity (92.3%), so discriminating analysis based on AFP, IL-6, and IL-10 had high diagnostic accuracy in discriminating HCC from non-HCC benign liver diseases, especially among patients with low (<20 ng/mL) AFP level, and these results were in agreement with the previous studies [42, 55, 56]. 

Another interesting finding in our study is that IL-6 and IL-10 values were correlated significantly with the tumor size which is consistent with results obtained by Malaguarnera et al. [42].

In our study there was a strong correlation between serum IL-6 and IL-10 levels in patients with LC and HCC and this agreed with Gastl et al. [57].

## 5. Conclusion

High levels of IL-6 and IL-10 are observed in HCC patients, correlate with tumor size, and may be helpful to identify a subset of HCC patients with low AFP level. Serum IL-6 and IL-10 levels may serve as complementary tumor markers and contribute to the differential diagnosis in HCC patients. 

## Figures and Tables

**Table 1:** Comparison between age, liver functions, and blood counts in the studied groups.

	Group	Range	Mean	S.E	*F* value	*P* value
Age (year)	Control	33–77	51.50	2.48	2.313	0.083
	HCV	33–71	55.05	2.02		
	LC	32–78	55.60	2.43		
	HCC	35–76	60.15	2.39		

T BIL (mg %)	Control	0.4–0.9	0.70^a^	0.03	5.061	0.003∗
	HCV	1–5.4	2.65^b^	0.32		
	LC	1.1–34	5.74^c^	1.80		
	HCC	1.5–6	2.72^b^	0.30		

D BIL (mg %)	Control	0.1–0.29	0.19^a^	0.09	6.238	0.001∗
	HCV	0.2–2.5	1.20^b^	0.16		
	LC	0.4–14.2	2.69^c^	0.82		
	HCC	0–1.9	0.72^b^	0.09		

AST (u/L)	Control	13–34	22.05^a^	1.27	14.673	0.000∗
	HCV	30–108	69.80^b^	4.36		
	LC	31–402	96.45^b^	20.21		
	HCC	80–300	133.35^c^	13.00		

ALT (u/L)	Control	11–31	17.35^a^	1.18	11.516	0.000∗
	HCV	32–176	92.90^c^	8.33		
	LC	12–255	75.25^b^	16.55		
	HCC	34–103	64.65^b^	4.19		

Alb (g/dL)	Control	2.8–5.1	4.01^b^	0.12	25.608	0.000∗
	HCV	1.9–3.6	2.76^a^	0.12		
	LC	1.3–3.9	2.53^a^	0.16		
	HCC	1.7–3.4	2.79^a^	0.12		

Proth (sec)	Control	13–16	14.05	0.20	1.983	0.124
	HCV	14–19	16.20	0.34		
	LC	11.7–67	18.95	2.82		
	HCC	14.2–19.6	15.80	0.43		

TLC (×10^3^/cmm)	Control	4–10.2	6.97	0.42	1.732	0.168
	HCV	3.1–16.2	8.40	0.70		
	LC	3–36.1	10.37	1.69		
	HCC	3–36.1	10.37	1.69		

HB (g/dL)	Control	12.1–15.7	13.72^c^	0.25	12.505	0.000∗
	HCV	6.1–13	9.90^a^	0.55		
	LC	4.1–16	10.09^a^	0.70		
	HCC	9–15	11.80^b^	0.39		

Hct (%)	Control	40–50	42^b^	0.01	188.062	0.000∗
	HCV	20–40	31^b^	0.01		
	LC	13–44.4	28.97^a^	1.70		
	HCC	13.3–44.4	28.97^a^	1.70		

Plat (×10^3^/cmm)	Control	150–450	262.10^b^	15.83	16.161	0.000∗
	HCV	73–431	233.95^b^	20.60		
	LC	48–331	161.60^a^	17.03		
	HCC	60–160	123.85^a^	6.43		

S.E: standard error.

^*^There is a significant difference between groups at *P* < 0.05.

The same letter means that there is no significant difference between each two groups at *P* < 0.05.

The different letters means that there is a significant difference between each two groups at *P* < 0.05.

**Table 2:** Comparison between AFP, IL-6, and IL-10 in the studied groups.

	Group	Range	Mean	S.E	*F*-value	*P*-value
AFP ng/mL	Control	0.6–8	3.335^a^	0.41	24.798	0.000∗
	HCV	2.4–45.7	13.51^b^	3.27		
	LC	2.7–120	21.245^c^	5.97		
	HCC	12–1000	255.8^d^	48.43		

IL-6 pg/mL	Control	0.9–7.5	2.57^a^	0.31	22.686	0.000∗
	HCV	1.4–14.9	5.78^b^	0.74		
	LC	3–11.2	7.49^b^	0.43		
	HCC	3.9–34.6	13.99^c^	1.80		

IL-10 pg/mL	Control	1.2–7.8	3.31^a^	0.42	18.549	0.000∗
	HCV	3.1–14.5	5.18^b^	0.60		
	LC	3.5–13.8	7.37^b^	0.53		
	HCC	3.3–42.1	13.69^c^	1.89		

The same letter means that there is no significant difference between each two groups at *P* < 0.05.

The different letters means that there is a significant difference between each two groups at *P* < 0.05.

**Table 3:** Diagnostic values of AFP, IL-6, and IL-10 for the detection of HCC.

	Cutoff value	AUC	Sensitivity	Specificity	Accuracy	+LR	−LR	PPV	NPV
AFP	>20 ng/mL	0.76	80	80	80%	4	0.25	57.1	92.3
IL-6	>8.6 pg/mL	0.93	90	86.67	87.5%	6.75	0.12	69.2	96.3
IL-10	>9.7 pg/mL	0.91	80	96.67	92.5%	24	0.21	88.9	93.5

+LR: positive likelihood ratio.

−LR: negative likelihood ratio.

PPV: positive predictive value.

NPV: negative predictive value.
